# Using environmental and exercise physiology to address gender inequalities in climate change and occupational health research

**DOI:** 10.1113/EP091456

**Published:** 2024-12-09

**Authors:** Rebekah A. I. Lucas

**Affiliations:** ^1^ School of Sport, Exercise and Rehabilitation Sciences University of Birmingham Birmingham UK; ^2^ La Isla Network Washington, DC USA

**Keywords:** climate change, exercise physiology, female, heat stress, performance, productivity, women, work

## Abstract

Climate change is a health‐risk and health‐inequity multiplier with excessive heat exposure a direct climate change impact already affecting the health and livelihood of billions globally. Women face greater risks and burdens from climate change impacts. Biological sex may or may not influence an individual's thermoregulatory capacity, heat tolerance or heat susceptibility. However at a population level, sex differences in physiological characteristics (anthropometrics, aerobic capacity, etc.) likely affect thermoregulatory capacity. Still, gender appears to play the most significant role in heat exposure and resulting health impacts. For climate change resilience and adaptation strategies to be effective, public health and occupational guidance/governance must be based on comprehensive and representative evidence. The current dearth of empirical evidence on how excessive heat exposure affects women prohibits this. Environmental and exercise physiology can help address this lack of empirical evidence by adhering to inclusive research guidelines. This paper is based on a symposium presentation given at Physiology 2023 in Harrogate, UK. Using a multi‐year cohort study on industrial agricultural workers (the Adelante Initiative) as a case study, this review discusses the role of environmental and exercise physiology in generating inclusive research and evidence to inform occupational and public health guidance/governance for climate change resilience and adaptation, specifically heat exposure.

## Abstract (highlights)

- **What is the topic of this review?**
  The role of environmental and exercise physiology in generating inclusive research and evidence to inform guidance/governance for climate change adaptation, specifically heat exposure.
- **What advances does it highlight?**
  There remains a lack of empirical evidence on how excessive heat exposure specifically affects women. The under‐representation of women in biomedical and sport‐related research, along with the clear health impacts of climate change, necessitates future research rapidly becoming more inclusive and climate‐sensitive.

## 1 THE PROBLEM

Climate change is the single biggest threat to global health worldwide. Excessive heat exposure is an immediate impact of climate change that is directly associated with adverse acute and chronic health effects (i.e., health illness/injury, kidney injury, heat stroke, adverse pregnancy outcomes, worsening sleep patterns and mental health, and aggravation of cardiovascular and respiratory diseases; Romanello et al., 2022). Excessive heat exposure also impairs health indirectly by restricting people's capacity to work and engage in physical activity (Lucas et al., 2014), undermining livelihoods and socioeconomic determinants of health (Day et al., 2019). This vulnerability is especially pronounced among outdoor workers and those engaging in physically demanding outdoor activities (Flouris et al., 2018), which in developing regions includes water collection and small‐scale farming (i.e., smallholding; Dimitrov, 2019). The deleterious impact of excessive heat exposure on labour capacity already presents a serious threat to billions of workers worldwide, with recent models estimating that loss of labour capacity due to heat exposure caused a global potential income loss of US$835 billion in 2023 (Romanello et al., 2024). By 2030 this is projected to reach US$2400 billion, equating to 80 million full‐time jobs, with a 1.5°C rise in global temperature by the end of this century (ILO, 2019). Notably, the agricultural sector is the most affected, contributing two‐thirds of all labour losses (ILO, 2019; Romanello et al., 2022). More broadly, the International Labour Organization (ILO) estimates that at least 2.41 billion workers worldwide are exposed to excessive heat annually, leading to nearly 23 million occupational injuries, 19,000 deaths and 2.09 million disability‐adjusted life years as a result of heat stress at work (ILO, 2024). Thus, occupational heat exposure is a serious economic and health climate change impact that is currently affecting the health and livelihood of workers globally, particularly agricultural workers.

Climate change‐related health impacts disproportionately affect the poorest and most vulnerable communities (Romanello et al., 2022). At a global level, women face greater risks and burdens from climate change impacts, with climate change a risk multiplier for gender‐based health disparities (Desai & Zhang, 2021; Sorensen et al., 2018). Globally, women's employment in agriculture matches men's (∼25% vs. ∼25%, respectively; ILO, 1991–2022). Regionally, women's employment in agriculture can reach as high as 20% in Latin America, 60% in South Asia and 68% in Sub‐Saharan Africa (ILO, 1991–2022; Patil & Babus, 2018), with agricultural workers in these regions highly susceptible to climate change health and productivity impacts, particularly those related to excessive heat stress (ILO, 2019; Romanello et al., 2022). Women's involvement in agriculture is diverse and complex, ranging from formal employment to unpaid work on family farms or other agricultural enterprises (Patil & Babus, 2018). Added to this, domestic water responsibilities (including household and smallholding water requirements) largely fall on women and girls in households without running water. It has been estimated that, as a global collective, women and girls *daily* spend up to 200 million hours collecting water (United Nations, 2016). With global warming, daily water collection times for women are projected to increase by 30% globally and up to 100% regionally under a high‐emission scenario (Carr et al., 2024). Such projections highlight the disproportionate burden climate change impacts have on women and girls in some regions. This has been increasingly recognised by intergovernmental bodies in recent years. Indeed, the Intergovernmental Panel on Climate Change (IPCC) Sixth Assessment Report (particularly the Working Group II report on Impacts, Adaptation and Vulnerability) includes more references to gender, equity and justice than any previous IPCC report (IPCC, 2022). Moreover, the recent ILO report states that women workers can face higher climate change‐related occupational health and safety risks due to their job (e.g., subsistence agriculture) and during different life stages (e.g., pregnancy) (ILO, 2024). Thus, at a governance level, there is an increasing acknowledgement of the need to integrate gender equity into climate change resilience and adaptation strategies.

**FIGURE 1:**
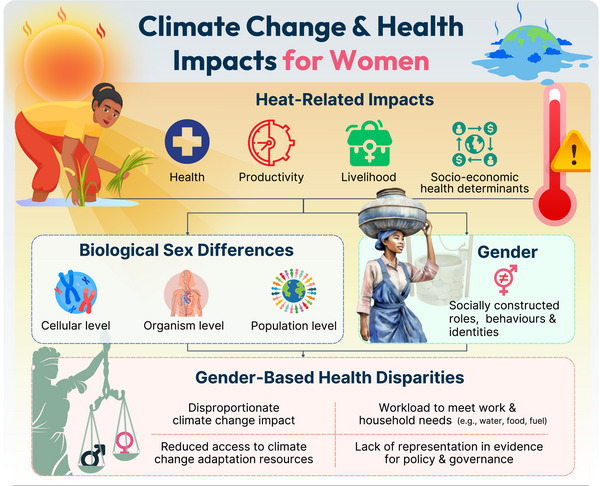
A conceptual framework for assessing climate change health impacts in women.

To address climate change and occupational gender inequalities, public health and occupational guidance/governance must be based on comprehensive and representative evidence. However, there remains a dearth of empirical evidence on how excessive heat exposure affects women. Systematic reviews of exercise thermoregulation and heat adaptation literature have shown women are significantly under‐represented, accounting for ≤30% of total participants (Hutchins et al., 2021; Kelly et al., 2024). This finding was echoed in our recent systematic scoping review investigating the impacts of heat stress on performance and productivity in females (Gilworth et al., 2024), which further highlighted a yawning gap in occupational‐focused evidence, as just 7% (3/41) of included studies specifically assessed women's performance or productivity in the heat within a work context (Sen et al., 1983; Tenaglia et al., 1999; Vincent et al., 2018). There are biological sex (the biological attributes of females and males; Rich‐Edwards et al., 2018) differences at the cell, organism and population level that affect thermoregulatory physiological response to heat stress (Giersch et al., 2022). However, the potential confounding influence of menstrual cycle phase can be accounted for (via study design or analytical approach) and this is no longer an indubitable rationale for excluding females from participant cohorts, even in thermoregulatory/heat‐focused research (Hutchins et al., 2021; Meyer & Cobley, 2024). To date there is no clear evidence that biological sex differentially affects aerobic performance in the heat (Gilworth et al., 2024) or exertional heat stroke susceptibility (Giersch et al., 2023, 2022), although physiological characteristics that at a population level differ between females and males (i.e., anthropometric characteristics, aerobic capacity and metabolic heat production; Giersch et al., 2022) have been shown to affect an individual's thermoregulatory capacity during exercise heat stress (Cottle et al., 2022; Wolf et al., 2023). Apart from biological sex differences, gender (encompassing socially constructed roles, behaviours and identities on a spectrum, including femininity and masculinity; Rich‐Edwards et al., 2018) appears to significantly influence heat exposure and its health impacts (see Figure 1). For example, poor access to healthcare and cooling facilities due to personal safety concerns and a lack of access to personal transportation, culturally prescribed heavy clothing garments that limit evaporative cooling, plus a lack of sanitation/welfare facilities all limit women's capacity to behaviourally adapt and increase their burden and risk of heat stress (Shanmugam et al., 2023; Sorensen et al., 2018). Thus, there remains substantial gaps in our understanding of how women perceive, behave and physically respond to excessive heat stress across various settings and life‐stages. While female‐specific, biological sex and gender‐related research questions/studies will address this, so too will the general inclusion of women within participant cohorts.

### 1.1 Occupational heat exposure in women, a case study

The Adelante Initiative (https://adelanteinitiative.org) is an ongoing multi‐stakeholder intervention (Rest, Shade, Hydration & Hygiene) that employs the PREP (Prevention Resilience Efficiency and Protection) research methodology, with the objective of reducing heat stress among sugarcane workers at Ingenio San Antonio (ISA), Chichigalpa, Nicaragua. Launched in 2017, this initiative was a response to the Central American epidemic of chronic kidney disease of non‐traditional origin (CKDnt), which has been characterized as an occupational disease driven by heat stress from strenuous manual labour performed in hot environments that is prevalent in young male agricultural workers, particularly sugarcane workers (Wesseling et al., 2020). At ISA 15% of the workforce are women (Hansson et al., 2024b). Very few women are employed as burned‐cane cutters (reported to be the most strenuous and difficult job by workers and managers; Lucas et al., 2023). However, women are increasingly employed in other types of strenuous outdoor manual work, such as seed cutting. In a small observational study we found women seed cutters worked at a higher physiological workload than their male counterparts and consequently experience a greater degree of heat strain (Lucas et al., 2023). Another study by our group found women workers had a lower sensitivity to heat‐related productivity loss (smaller percentage productivity decline from <28 to >31 WBGT°C) compared to male counterparts, when examining the impact of environmental heat stress on productivity across a 5‐year period (Hansson et al., 2024a). This may be due to women workers having a lower absolute productivity rate than their male counterparts. Alternatively, it may be attributed to behavioural factors, such as women workers adopting better pacing strategies, which is also demonstrated in female non‐elite marathon runners (Trubee et al., 2014). Of note, at ISA's hospital 95% of AKI cases are men (Hansson et al., 2024b). We have also previously shown a clear disproportionate male preponderance in serum creatinine increases across a harvest period among asymptomatic seed cutters (≥1.5 times the baseline value, indicating reduce kidney function; Glaser et al., 2020). Collectively these studies demonstrate that women do perform physically demanding jobs within the sugarcane industry and thus are exposed to excessive levels of occupational heat stress. However, they (as yet) do not frequently perform jobs/tasks (i.e., burned‐cane cutting) associated with the greatest CKDnt risk in this population. From the evidence to date it could be suggested that kidney injury risk is lower in women working in industrial sugarcane as compared to men in this industry. However, given the significantly higher ratio of men working at ISA, particularly in jobs with the highest heat exposure levels, potential biological sex or gender differences should be viewed with caution. Further to this, community‐based studies indicate an increased risk for reduced kidney function in female agricultural workers (albeit lower than male agricultural workers; Lebov et al., 2015). Therefore, although female industrial sugarcane workers in this region have lower rates of AKI, impaired kidney function and CKDnt, it is unclear if this is due to lower exposure rates, biological sex and gender differences (as with cardiovascular health and disease; Regitz‐Zagrosek & Gebhard, 2023), fewer women working in industrial agricultural or a combination of these factors. Research that focuses on women in susceptible workforces such as industrial sugarcane workers provides an important opportunity to assess biological sex differences in heat‐related injuries/diseases such as CKDnt, which could inform injury/disease aetiology. More critically, workplace interventions aiming to address excessive heat exposure must consider all potential at‐risk workers and implement effective interventions for all (Shanmugam et al., 2023; Sorensen et al., 2018).

## 2 LESSONS WE NEED TO LEARN

The lack and consequence therein of females’ and women's under‐representation in biomedical and sport & exercise‐related research are well established. Similarly, the health impacts of climate change (or rather the climate crisis) are clear and resounding. Both scenarios require catalysed research to rapidly generate *inclusive* and *climate‐sensitive* health action. Physiologists play a role in achieving both these aims. Physiological research studies must include both male and female participants, aiming for gender balance via study design and recruitment (unless there is a specific reason for single‐gender studies; Meyer & Cobley, 2024). Moreover, detailed reporting of participant characteristics is necessary, including menstrual cycle phase and hormonal contraceptive use, etc. (for further recommendations see Janse De Jonge et al., 2019). Improving practice in these areas will reduce research bias, improve the applicability and reproducibility of research evidence, and thus enhance overall research quality and impact (UKRI, 2022). Physiologists also have a significant role to play in climate change health research and action, in terms of climate change resilience, adaptation as well as the co‐benefits associated with climate change mitigation. There are increasing opportunities for interdisciplinary climate change and health research to achieve this. However, we must avoid perpetuating historical biases by continuing to use evidence‐based on cisgender males as representative of the majority in public and occupational health. We also cannot afford to recommend adaptation or lifestyle strategies that do not consider climate change mitigation and social‐cultural factors (Figure 1).

## 3 CONCLUSION

Occupational heat exposure presents a significant global economic and health challenge, particularly for agricultural workers. Women may be disproportionately affected, and there is a need to integrate gender equity into climate change strategies. However, there remains a lack of empirical evidence on how excessive heat exposure specifically affects women. Biological sex and gender both appear to influence heat exposure and resulting health impacts. Emerging research from our group has shown that women in the sugarcane industry are exposed to significant occupational heat stress. Females in this context also appear to have lower rates of AKI and impaired kidney function, but it remains unclear whether this is due to lower exposure or biological sex differences. Further research is essential to understand these dynamics and inform effective workplace interventions. Ultimately, workplace strategies must consider all at‐risk workers to mitigate the impacts of excessive heat exposure comprehensively. The under‐representation of women in biomedical and sport‐related research, along with the clear health impacts of climate change, necessitates future research rapidly becoming more inclusive and climate‐sensitive. Where possible, physiologists must ensure gender balance when designing research studies and report female participant characteristics in sufficient detail to reduce bias and improve research quality. We must also stop perpetuating historical biases when designing or informing climate change health research/recommendations. To best protect people, livelihoods and economies, an inclusive climate‐focused research agenda is needed to generate comprehensive and representative public and occupational health recommendations and governance.

## AUTHOR CONTRIBUTIONS

Sole author.

## CONFLICT OF INTEREST

None declared.

## FUNDING INFORMATION

No funding was received for this work.
